# Association Between Mode of Delivery and Postpartum Depression: A Cross-Sectional Study in Saudi Arabia

**DOI:** 10.7759/cureus.47013

**Published:** 2023-10-14

**Authors:** Yousra Alturki, Samia Badea, Orjwan Kasmi, Lujain Alhashmi, Tarek Arab

**Affiliations:** 1 College of Medicine, Umm Al-Qura University, Makkah, SAU; 2 Obstetrics and Gynaecology, King Faisal Specialist Hospital and Research Centre, Madinah, SAU

**Keywords:** edinburgh postnatal depression scale, public health, mode of delivery, major depression, postpartum depressions

## Abstract

Background

This study was conducted to explore the association between postpartum depression (PPD) and mode of delivery in pregnant women.

Methods

This cross-sectional study was conducted in the western region of Saudi Arabia, among 173 women from the general population who met the inclusion criteria and participated in the study from April to September 2022. The Edinburgh Postnatal Depression Scale (EPDS) was used to assess PPD. Statistical analysis was performed using R version 3.6.3 (R Foundation for Statistical Computing, Vienna, Austria). Counts and percentages were used for categorical variables, and means ± standard deviation were used for continuous variables. Hypothesis testing was done at a significance level of 5%.

Results

It was reported by 59.5% of the respondents to be having depression. Compared to respondents who reported elective cesarean or natural delivery, those who reported emergency cesarean delivery had significantly higher average EPDS scores (p = 0.036). Positive correlations were found between depression scores and all Postpartum Bonding Questionnaire (PBQ) subscales, suggesting that higher PBQ subscale scores were associated with a greater risk of depression as determined by the EPDS questionnaire. In addition, a higher prevalence of PPD was associated with the presence of chronic illnesses (p = 0.016).

Conclusion

Our study indicated that although there was no correlation between mode of delivery and PPD, emergency cesarean section could contribute to PPD. Furthermore, other factors such as chronic illness and educational level can affect the risk of PPD.

## Introduction

Postpartum depression (PPD) is a form of major depression that begins within the first four weeks of birth. Some women experience complex, mixed changes after childbirth, with some being associated with PPD (e.g., chemical, psychological, and social changes). The chemical change involves a rapid and sharp drop in hormone levels (i.e., estrogen and progesterone), which return to pre-pregnancy levels. In addition, psychological and social changes after having a baby increase the risk of PPD [1]. Several studies have estimated the prevalence of PPD in the Saudi Arabian cities of Dammam, Riyadh, and Jeddah to be 17.8%, 33.2%, and 20.9%, respectively [2-4].

The diagnostic criteria of PPD include low motivation, depressed mood, anhedonia, sleep disturbance, appetite irregularities, decreased attention, psychomotor disturbances, exhaustion, feelings of guilt or worthlessness, and thoughts of suicide, all of which must occur within two weeks after giving birth and indicate a change in previous function. These symptoms must produce clinically serious distress or impaired function that is not induced by drugs or another medical condition. Moreover, anxiety, overload, irritability, mood lability, and obsessional worry or preoccupation (often concerning the baby’s health, feeding, and bathing safety) are also common PPD symptoms. Suicidal thoughts are frequent in women with PPD, affecting around 20% of patients. PPD in women can also lead to thoughts of harming their children, and concerns regarding self-harm or newborn harm must be evaluated with a prompt referral for psychiatric assessment and care. Depressive symptoms within the first year after childbirth that may not match all of the criteria for a major depressive episode could still have a significantly negative impact on mothers, their children, and their families, and appropriate intervention may be required [5]. PPD involves multiple risk domains encompassing psychological, obstetric, biological, hormonal, social, and lifestyle risk factors.

PPD also has important implications for the mother’s mental health, quality of life, and interactions with their children, partner, and relatives; eventually, it may destroy the mother-child relationship, which is essential for children’s development. Mothers’ affection, sensitivity, and parenting styles are important for the healthy maturation of infants’ social, cognitive, and behavioral skills, and depressed mothers often show less attachment, less sensitivity, and sharper or more confusing parenting behavior, with reports of negative outcomes in children of depressed mothers [6]. A previous systematic review and meta-analysis showed that there was a significant association between the occurrence of PPD and mode of delivery [7]; this result is contrary to what other studies found, where the mode of delivery had no direct impact on the risk of developing PPD [8,9].

However, Eckerdal et al. found that women who deliver in an emergency setting either by emergency cesarean section (EMCS) or vacuum extraction constitute a high-risk group for PPD, and that fear of delivery and a history of depression might increase the odds of PPD [8]. Our aim in this study was to explore the association between PPD and the mode of delivery in Saudi Arabia. 

## Materials and methods

This cross-sectional, descriptive study was reviewed by the scientific board, and ethical approval was obtained from Umm Al Qura University, Makkah, Saudi Arabia with approval number HAPO-02-K-012-2022-03-1031. The Edinburgh Postnatal Depression Scale (EPDS) questionnaire was used to assess PPD in this study. The questionnaire comprises 10 Likert-scale items, each of which is rated on a scale from 0 to 3, with a maximum possible score of 30. The total score is categorized based on previous recommendations: 0-9 represents a non-depressed case, 10-12 represents a borderline case, and a score of 13 or more represents a significant depression.

The Postpartum Bonding Questionnaire (PBQ) has 25 statements, each followed by six alternative responses ranging from “always” to “never.” Positive responses, such as “I enjoy playing with my baby,” are scored from 0 (“always”) to 5 (“never”); negative responses, such as “I am afraid of my baby,” are scored from 5 (“always”) to 0 (“never”). The scores are summated for each factor, with a high score indicating pathology [10-12].

PBQ is divided into four main factors, each of them measuring an aspect related to bonding [13]: (1) impaired bonding (Q: 1, 2, 6, 7, 8, 9, 10, 12, 13, 15, 16, 17), with a cutoff score of 11 (i.e., scores higher than 11 indicate bonding disorder); (2) rejection and pathological anger (Q: 3, 4, 5, 11, 14, 21, 23), with a cutoff score 16; (3) anxiety about the infant (AI; Q: 19, 20, 22, 25), with a cutoff score of 9; and (4) incipient abuse (IA; Q: 18, 24), with a cutoff score of 2.

The Short Form-8 (SF-8) was used to assess the health-related quality of life in the included respondents; it includes eight Likert-scale items with scores ranging from 5 (“never” ) to 1 (“very severe”), with higher scores indicating a better quality of life. The two-factor model (physical and mental health) was a good fit for the SF-8 data.

Statistical analysis 

Statistical analysis was performed using R version 3.6.3 (R Foundation for Statistical Computing, Vienna, Austria). Counts and percentages were used to summarize categorical variables. The mean ± standard deviation was used to summarize continuous variables. Spearman’s correlation was used to assess the association between SF-8 and EPDS total and subscale scores. A chi-squared test of independence was used to assess the association between categorical variables. Binary logistic regression was used to assess factors associated with depression. Factors associated with depression at the 0.1 level according to a univariate analysis were included in the model. Hypothesis testing was performed at a level of significance of 5%.

## Results

The study sample included 173 females [14]. The majority were married (94.8%). Regarding maternity status, approximately more than two-thirds of the respondents were 8-16 weeks postpartum (70.5%), and approximately one-third were 2-4 weeks postpartum (29.5%). Less than 10% reported having chronic diseases and 3.47% reported psychiatric diseases. Postpartum depression was diagnosed in 14.5% of the included respondents. Two-thirds of the respondents were living in Mecca (61.3%). Regarding education, more than three-quarters of the respondents had a bachelor’s degree (78%), and only 18.5% had completed high school. Only one-third of the respondents were employed, and more than three-quarters reported that the monthly income was sufficient (78%) ( Table 1). 

**Table 1:** Descriptive statistics for the study sample Data were summarized using counts and percentages.

Variables		N (%)
Marital status	Married	164 (94.8%)
	Widowed/divorced	9 (5.20%)
Age (years)	18-30	65 (37.6%)
	30-50	98 (56.6%)
	50-64	10 (5.78%)
Maternity status	2-4 weeks postpartum	51 (29.5%)
	8-16 weeks postpartum	122 (70.5%)
Chronic diseases	No	156 (90.2%)
	Yes	17 (9.83%)
Psychiatric diseases	No	167 (96.5%)
	Yes	6 (3.47%)
Ever diagnosed with postpartum depression	No	148 (85.5%)
	Yes	25 (14.5%)
Residency	Jeddah	47 (27.2%)
	Mecca	106 (61.3%)
	Taif	20 (11.6%)
Education	College	135 (78.0%)
	High school	32 (18.5%)
	Illiterate	1 (0.58%)
	Middle school	4 (2.31%)
	Primary school	1 (0.58%)
Monthly income	Enough	135 (78.0%)
	Not enough	38 (22.0%)
Housing	Owner	95 (54.9%)
	Renter	78 (45.1%)
Employment	Employed	56 (32.4%)
	Student	15 (8.67%)
	Unemployed	102 (59.0%)

The number of previous pregnancies ranged from one (32.4%) to three or more (45.7%). Epidural anesthesia was used in three-quarters of the cases (71.1%). Gestational age was 37 weeks or more in 87.3% of the respondents (Table 2). 

**Table 2:** Pregnancy-related characteristics of the included respondents Data were summarized using counts and percentages.

Variables		N (%)
Parity (previous pregnancies)	1	56 (32.4%)
	2	38 (22.0%)
	3+	79 (45.7%)
Previous delivery	Elective cesarean	21 (12.1%)
	Emergency cesarean	43 (24.9%)
	Natural	109 (63.0%)
Gestational age	<37 weeks	22 (12.7%)
	≥37 weeks	151 (87.3%)
Anesthesia	Epidural	123 (71.1%)
	General	50 (28.9%)
Feeding	Bottle feeding	35 (20.2%)
	Breastfeeding	138 (79.8%)

Responses were coded so that lower scores represent less depression.

**Figure 1:**
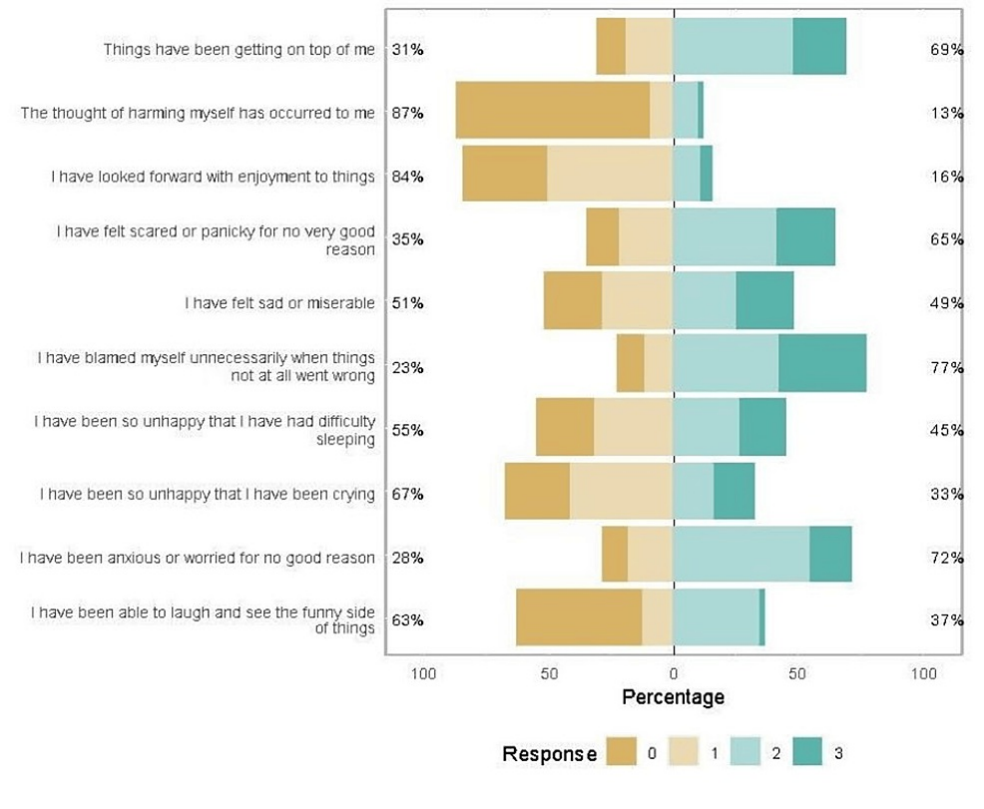
Summary of responses to Edinburgh Postnatal Depression Scale (EPDS) questions

The EPDS results indicated a high prevalence of depression, as demonstrated by the higher score for the majority of the questions (Figure 1). Three-quarters of the respondents were anxious or worried for no good reason “very often” or “sometimes.” Similarly, three-quarters of the respondents blamed themselves “most of the time” or “sometimes” when things went wrong.

Responses were recorded that higher scores represent a higher degree of agreement.

**Figure 2:**
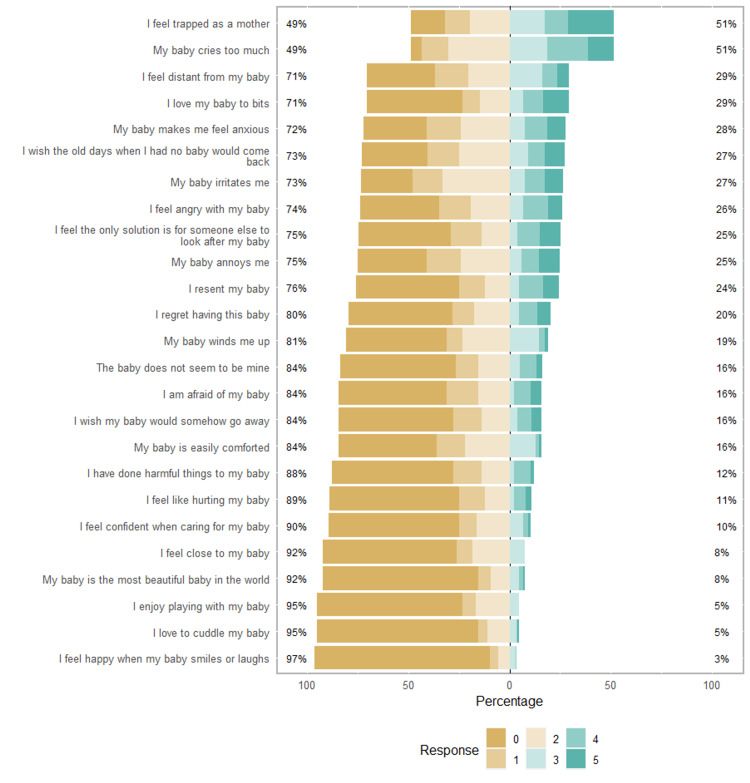
Summary of responses to the Postpartum Bonding Questionnaire (PBQ).

Half of the included mothers thought that their children “often” or “always” cried a lot, and the same percentage felt they were “often” or “always” trapped as a mother (Figure 2).

A statistically significant association was observed between high rejection and pathological anger (RPA) score and age, with respondents aged 50-64 years showing a higher prevalence of high RPA scores (p = 0.014). A high RPA score was also more prevalent in widowed/divorced respondents than married respondents (55.6% vs. 18.9%, respectively, p = 0.02). Neither chronic nor psychiatric disorders were significantly associated with any of the PBQ subscales. College education was associated with a lower prevalence of high RPA and Al scores than respondents with lower education (p values of 0.002 and 0.003, respectively). Low monthly income was associated with a higher incidence of high RPA (p = 0.011). Neither employment, housing nor gravidity showed a statistically significant association with any of the PBQ subscales. Natural delivery was associated with a lower incidence of a high AI score. Finally, gestational age, anesthesia, and feeding were not associated with any of the PBQ subscales (Table 3). 

**Table 3:** Sociodemographic, pregnancy, and maternity-related factors associated with Postpartum Bonding Questionnaire (PBQ) subscales IB: impaired bonding (Q: 1, 2, 6, 7, 8, 9, 10, 12, 13, 15, 16, 17), with a cutoff score of 11 (i.e., scores above 11 indicate bonding disorder). RPA: rejection and pathological anger (Q: 3, 4, 5, 11, 14, 21, 23), with a cutoff score of 16. AI: anxiety about the infant (Q: 19, 20, 22, 25), with a cutoff score of 9. IA: incipient abuse (Q: 18, 24), with a cutoff score of 2. Analysis was performed using a chi-squared test of independence.

Variables	IB			RPA			AI			IA		
	Low	High	p-Value	Low	High	p-Value	Low	High	p-Value	Low	High	p-Value
	N = 60	N = 113		N = 137	N = 36		N = 148	N = 25		N = 54	N = 119	
Age (years):			0.750			0.014			0.136			0.243
18-30	25 (38.5%)	40 (61.5%)		54 (83.1%)	11 (16.9%)		59 (90.8%)	6 (9.23%)		25 (38.5%)	40 (61.5%)	
30-50	32 (32.7%)	66 (67.3%)		79 (80.6%)	19 (19.4%)		82 (83.7%)	16 (16.3%)		27 (27.6%)	71 (72.4%)	
50-64	3 (30.0%)	7 (70.0%)		4 (40.0%)	6 (60.0%)		7 (70.0%)	3 (30.0%)		2 (20.0%)	8 (80.0%)	
Marital status:			0.165			0.020			0.124			0.276
Married	59 (36.0%)	105 (64.0%)		133 (81.1%)	31 (18.9%)		142 (86.6%)	22 (13.4%)		53 (32.3%)	111 (67.7%)	
Widowed/divorced	1 (11.1%)	8 (88.9%)		4 (44.4%)	5 (55.6%)		6 (66.7%)	3 (33.3%)		1 (11.1%)	8 (88.9%)	
Chronic diseases:			0.454			1.000			0.716			0.657
No	56 (35.9%)	100 (64.1%)		123 (78.8%)	33 (21.2%)		134 (85.9%)	22 (14.1%)		50 (32.1%)	106 (67.9%)	
Yes	4 (23.5%)	13 (76.5%)		14 (82.4%)	3 (17.6%)		14 (82.4%)	3 (17.6%)		4 (23.5%)	13 (76.5%)	
Psychiatric diseases:			0.666			1.000			1.000			0.667
No	59 (35.3%)	108 (64.7%)		132 (79.0%)	35 (21.0%)		143 (85.6%)	24 (14.4%)		53 (31.7%)	114 (68.3%)	
Yes	1 (16.7%)	5 (83.3%)		5 (83.3%)	1 (16.7%)		5 (83.3%)	1 (16.7%)		1 (16.7%)	5 (83.3%)	
Residency:			0.606			0.052			1.000			0.511
Jeddah	19 (40.4%)	28 (59.6%)		36 (76.6%)	11 (23.4%)		40 (85.1%)	7 (14.9%)		15 (31.9%)	32 (68.1%)	
Mecca	34 (32.1%)	72 (67.9%)		89 (84.0%)	17 (16.0%)		91 (85.8%)	15 (14.2%)		35 (33.0%)	71 (67.0%)	
Taif	7 (35.0%)	13 (65.0%)		12 (60.0%)	8 (40.0%)		17 (85.0%)	3 (15.0%)		4 (20.0%)	16 (80.0%)	
Education:			0.110			0.002			0.003			0.351
College	53 (39.3%)	82 (60.7%)		113 (83.7%)	22 (16.3%)		122 (90.4%)	13 (9.63%)		47 (34.8%)	88 (65.2%)	
High school	7 (21.9%)	25 (78.1%)		23 (71.9%)	9 (28.1%)		22 (68.8%)	10 (31.2%)		7 (21.9%)	25 (78.1%)	
Illiterate	0 (0.00%)	1 (100%)		0 (0.00%)	1 (100%)		1 (100%)	0 (0.00%)		0 (0.00%)	1 (100%)	
Middle school	0 (0.00%)	4 (100%)		1 (25.0%)	3 (75.0%)		3 (75.0%)	1 (25.0%)		0 (0.00%)	4 (100%)	
Primary school	0 (0.00%)	1 (100%)		0 (0.00%)	1 (100%)		0 (0.00%)	1 (100%)		0 (0.00%)	1 (100%)	
Monthly income:			0.028			0.011			0.996			0.084
Enough	53 (39.3%)	82 (60.7%)		113 (83.7%)	22 (16.3%)		116 (85.9%)	19 (14.1%)		47 (34.8%)	88 (65.2%)	
Not enough	7 (18.4%)	31 (81.6%)		24 (63.2%)	14 (36.8%)		32 (84.2%)	6 (15.8%)		7 (18.4%)	31 (81.6%)	
Employment:			0.597			0.082			1.000			0.795
Employed	22 (39.3%)	34 (60.7%)		48 (85.7%)	8 (14.3%)		48 (85.7%)	8 (14.3%)		16 (28.6%)	40 (71.4%)	
Student	4 (26.7%)	11 (73.3%)		14 (93.3%)	1 (6.67%)		13 (86.7%)	2 (13.3%)		4 (26.7%)	11 (73.3%)	
Unemployed	34 (33.3%)	68 (66.7%)		75 (73.5%)	27 (26.5%)		87 (85.3%)	15 (14.7%)		34 (33.3%)	68 (66.7%)	
Housing:			0.886			1.000			0.333			0.298
Owner	32 (33.7%)	63 (66.3%)		75 (78.9%)	20 (21.1%)		84 (88.4%)	11 (11.6%)		26 (27.4%)	69 (72.6%)	
Renter	28 (35.9%)	50 (64.1%)		62 (79.5%)	16 (20.5%)		64 (82.1%)	14 (17.9%)		28 (35.9%)	50 (64.1%)	
Parity:			0.113			0.775			0.786			0.183
1	20 (35.7%)	36 (64.3%)		46 (82.1%)	10 (17.9%)		49 (87.5%)	7 (12.5%)		18 (32.1%)	38 (67.9%)	
2	18 (47.4%)	20 (52.6%)		29 (76.3%)	9 (23.7%)		33 (86.8%)	5 (13.2%)		16 (42.1%)	22 (57.9%)	
3+	22 (27.8%)	57 (72.2%)		62 (78.5%)	17 (21.5%)		66 (83.5%)	13 (16.5%)		20 (25.3%)	59 (74.7%)	
Previous delivery:			0.461			0.384			0.030			0.655
Elective cesarean	9 (42.9%)	12 (57.1%)		18 (85.7%)	3 (14.3%)		16 (76.2%)	5 (23.8%)		7 (33.3%)	14 (66.7%)	
Emergency cesarean	12 (27.9%)	31 (72.1%)		31 (72.1%)	12 (27.9%)		33 (76.7%)	10 (23.3%)		11 (25.6%)	32 (74.4%)	
Natural	39 (35.8%)	70 (64.2%)		88 (80.7%)	21 (19.3%)		99 (90.8%)	10 (9.17%)		36 (33.0%)	73 (67.0%)	
Gestational age:			0.370			0.574			1.000			0.421
37 weeks	10 (45.5%)	12 (54.5%)		19 (86.4%)	3 (13.6%)		19 (86.4%)	3 (13.6%)		9 (40.9%)	13 (59.1%)	
≥37 weeks	50 (33.1%)	101 (66.9%)		118 (78.1%)	33 (21.9%)		129 (85.4%)	22 (14.6%)		45 (29.8%)	106 (70.2%)	
Anesthesia:			0.516			0.091			0.278			0.137
Epidural	45 (36.6%)	78 (63.4%)		102 (82.9%)	21 (17.1%)		108 (87.8%)	15 (12.2%)		43 (35.0%)	80 (65.0%)	
General	15 (30.0%)	35 (70.0%)		35 (70.0%)	15 (30.0%)		40 (80.0%)	10 (20.0%)		11 (22.0%)	39 (78.0%)	
Feeding:			0.148			0.571			0.438			0.071
Bottle feeding	8 (22.9%)	27 (77.1%)		26 (74.3%)	9 (25.7%)		28 (80.0%)	7 (20.0%)		6 (17.1%)	29 (82.9%)	
Breastfeeding	52 (37.7%)	86 (62.3%)		111 (80.4%)	27 (19.6%)		120 (87.0%)	18 (13.0%)		48 (34.8%)	90 (65.2%)	

**Figure 3:**
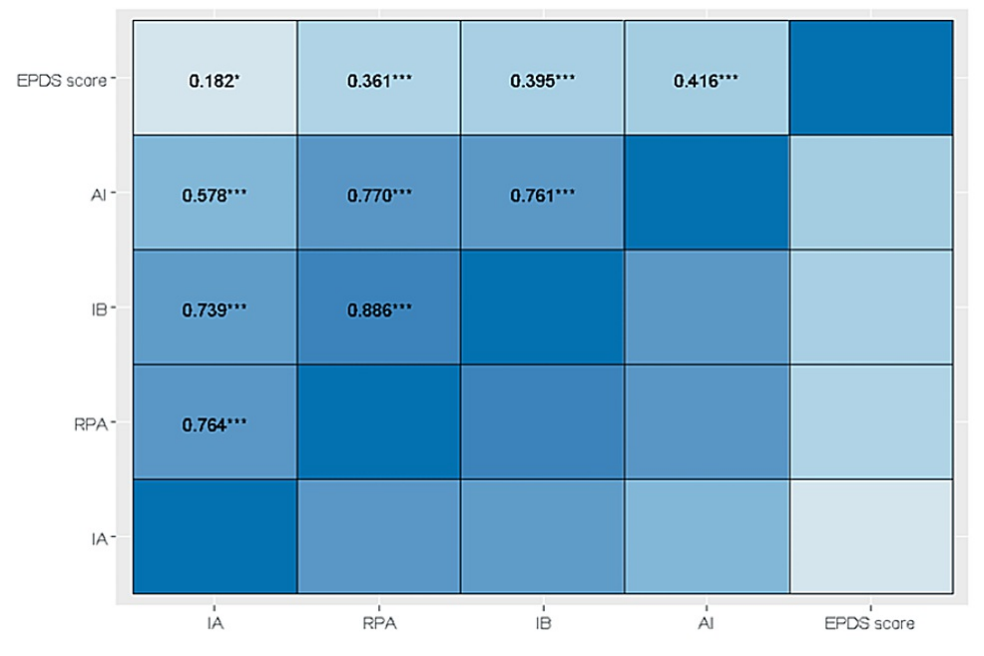
Association between EPDS and PBQ scores. Analysis was performed using Pearson’s correlation AI: anxiety about the infant; IB: impaired bonding; RPA: rejection and pathological anger; IA: incipient abuse EPDS: Edinburgh Postnatal Depression Scale; PBQ: Postpartum Bonding Questionnaire. *p < 0.05, **p < 0.01, ***p < 0.001.

A positive association was observed between depression scores and all subscales of the PBQ, indicating that higher scores on the PBQ subscales were associated with a higher risk of depression, as assessed using the EPDS questionnaire. The highest correlation was observed between EPDS and AI (r = 0.416, p < 0.001), whereas the lowest correlation was observed between EPDS and IA (r = 0.182, p < 0.05) (Figure 3). 

Based on the EPDS score, depression was present in 59.5% of the respondents. The average EPDS score was significantly higher in respondents who reported emergency cesarean than in those who reported elective cesarean or natural delivery (p = 0.036). High AI score was more prevalent in respondents who used CS for their last delivery than respondents who reported natural delivery (p = 0.03) (Table 4). 

**Table 4:** Association between delivery method, depression, and postpartum bonding Categorical data were summarized using counts and percentages. Continuous variables were summarized using the mean (SD). Continuous variables were compared using one-way ANOVA, and categorical variables were compared using the chi-square test of independence. AI: anxiety about the infant; IB: impaired bonding; RPA: rejection and pathological anger; IA: incipient abuse; ANOVA: analysis of variance.

EPDS score	All	Elective cesarean	Emergency cesarean	Natural	p-Value
	N = 173	N = 21	N = 43	N = 109	
	13.6 (5.83)	12.3 (5.08)	15.5 (5.73)	13.1 (5.88)	0.036
Depression category:					0.193
<10	43 (24.9%)	6 (28.6%)	8 (18.6%)	29 (26.6%)	
10-12	27 (15.6%)	3 (14.3%)	3 (6.98%)	21 (19.3%)	
>12	103 (59.5%)	12 (57.1%)	32 (74.4%)	59 (54.1%)	
IB	16.7 (10.8)	15.2 (11.8)	19.2 (11.7)	16.0 (10.1)	0.202
AI	4.76 (3.83)	4.95 (4.89)	5.88 (3.95)	4.28 (3.49)	0.063
IA	1.71 (2.66)	1.00 (2.05)	2.33 (3.05)	1.61 (2.57)	0.138
RPA	8.67 (7.60)	8.24 (7.58)	10.1 (7.96)	8.18 (7.46)	0.357
IB category:					0.461
Low	60 (34.7%)	9 (42.9%)	12 (27.9%)	39 (35.8%)	
High	113 (65.3%)	12 (57.1%)	31 (72.1%)	70 (64.2%)	
AI category:					0.030
Low	148 (85.5%)	16 (76.2%)	33 (76.7%)	99 (90.8%)	
High	25 (14.5%)	5 (23.8%)	10 (23.3%)	10 (9.17%)	
IA category:					0.655
Low	54 (31.2%)	7 (33.3%)	11 (25.6%)	36 (33.0%)	
High	119 (68.8%)	14 (66.7%)	32 (74.4%)	73 (67.0%)	
RPA category:					0.384
Low	137 (79.2%)	18 (85.7%)	31 (72.1%)	88 (80.7%)	
High	36 (20.8%)	3 (14.3%)	12 (27.9%)	21 (19.3%)	

The univariate analysis showed that the presence of chronic diseases was associated with a higher prevalence of depression (p = 0.023). Depression was also more prevalent in students (93.3%) than employed or unemployed respondents (p = 0.014). The number of previous pregnancies was also associated with the risk of depression (p = 0.027), as was the method of delivery, although the latter association was only statistically significant at the 0.1 level.

After using binary logistic regression to adjust for multiple confounders, the presence of chronic diseases retained its association with depression (OR = 7.13, p < 0.05). The odds of depression were 11 times higher in students than in employed respondents (OR = 11.04, p < 0.05). The association between a number of previous pregnancies and depression was no longer significant while the odds of depression were higher in emergency cesarean delivery than natural delivery (OR = 2.61, p < 0.05) (Table 5). 

**Table 5:** Sociodemographic and maternity factors associated with depression Categorical data were summarized using counts and percentages. Univariate analysis was performed using the chi-square test of independence. Multivariate analysis was performed using binary logistic regression. Respondents with no depression and borderline cases were combined prior to the analysis.

Variables	Univariate analysis			Multivariate analysis		
	No depression	Depression	p-Value	OR	95% CI	p-Value
	N = 70	N = 103				
Age (years):			0.717			
18-30	28 (43.1%)	37 (56.9%)				
30-50	39 (39.8%)	59 (60.2%)				
50-64	3 (30.0%)	7 (70.0%)				
Marital status:			0.315			
Married	68 (41.5%)	96 (58.5%)				
Widowed/divorced	2 (22.2%)	7 (77.8%)				
Chronic diseases:			0.023			
No	68 (43.6%)	88 (56.4%)		Ref.		
Yes	2 (11.8%)	15 (88.2%)		7.13	1.79-48.23	0.014
Psychiatric diseases:			0.082			
No	70 (41.9%)	97 (58.1%)				
Yes	0 (0.00%)	6 (100%)				
Residency:			0.731			
Jeddah	21 (44.7%)	26 (55.3%)				
Mecca	42 (39.6%)	64 (60.4%)				
Taif	7 (35.0%)	13 (65.0%)				
Education:			0.144			
College	60 (44.4%)	75 (55.6%)				
High school	8 (25.0%)	24 (75.0%)				
Illiterate	0 (0.00%)	1 (100%)				
Middle school	2 (50.0%)	2 (50.0%)				
Primary school	0 (0.00%)	1 (100%)				
Monthly income:			0.147			
Enough	59 (43.7%)	76 (56.3%)				
Not enough	11 (28.9%)	27 (71.1%)				
Employment:			0.014			
Employed	22 (39.3%)	34 (60.7%)		Ref		
Student	1 (6.67%)	14 (93.3%)		11.04	1.74-218.47	0.032
Unemployed	47 (46.1%)	55 (53.9%)		0.84	0.41-1.71	0.633
Housing:			0.546			
Owner	36 (37.9%)	59 (62.1%)				
Renter	34 (43.6%)	44 (56.4%)				
Parity:			0.027			
1	17 (30.4%)	39 (69.6%)				
2	22 (57.9%)	16 (42.1%)		0.52	0.20-1.36	0.186
3+	31 (39.2%)	48 (60.8%)		0.94	0.41-2.13	0.873
Previous delivery:			0.070			
Elective cesarean	9 (42.9%)	12 (57.1%)		1.28	0.46-3.58	0.637
Emergency cesarean	11 (25.6%)	32 (74.4%)		2.61	1.15-6.21	0.024
Natural	50 (45.9%)	59 (54.1%)		Ref.		
Gestational age:			1.000			
<37 weeks	9 (40.9%)	13 (59.1%)				
≥37 weeks	61 (40.4%)	90 (59.6%)				
Anesthesia:			0.106			
Epidural	55 (44.7%)	68 (55.3%)				
General	15 (30.0%)	35 (70.0%)				
Feeding:			0.072			
Bottle feeding	9 (25.7%)	26 (74.3%)		Ref.		
Breastfeeding	61 (44.2%)	77 (55.8%)		0.54	0.21-1.33	0.191

## Discussion

This study aimed to understand the relationship between the mode of delivery and PPD. The results showed that, between 2 and 16 weeks following delivery, there was no association between the type of delivery and depression symptoms. Nearly half of the respondents reported having depression. Compared to respondents who reported elective cesarean or normal delivery, women who reported emergency cesarean received significantly higher scores, which is in agreement with previous studies [8,15].

In contrast, a different study stated that the likelihood of PPD was not directly impacted by the mode of delivery; there was no difference in the incidence of depression months after delivery between cesarean and vaginal deliveries, regardless of whether they were complicated by perineal laceration or episiotomy [9,16].

However, another study showed that the occurrence of mild PPD was significantly associated with the mode of delivery. Women who delivered by CS had a 33% higher risk of mild PPD than those who delivered by vaginal delivery (VD), whereas women who delivered by EMCS had a 53% higher risk than those who delivered by spontaneous VD [7].

Similarly, in a study conducted in Japan on 89,954 women, those who underwent CS delivery were considered a target for PPD monitoring [17]; the same conclusion was also suggested by Heba Kamal Meky in their cross-sectional study [15]. A previous study conducted in Taiwan reported that CS was linked to an increased risk of PPD regardless of whether the women had a history of depression [18].

Another study in Riyadh showed a significant relationship between PPD and CS [3].

In our study, EMCS delivery was found to increase the risk of PPD. There are similarities between the findings expressed by our study and those described by a longitudinal study in Sweden [8], a systematic review, and a meta-analysis in China [7], all of which showed that women who delivered by EMCS were at high risk for PPD.

However, the findings of the current study about chronic disease do not agree with those of previous studies. For example, a Lebanese study found relationships between PPD and the level of education, unemployment, and chronic health problems [19]. In contrast, in our study, depression was more prevalent in students than in those who were employed or unemployed, which is consistent with the literature [20].

The limitation of our study was a small sample. The subjects were enrolled at different ages, and their clinical characteristics were not comparable in some of them due to already existing chronic diseases. 

To the best of our knowledge, no earlier studies of this type have been conducted among women in the western region of Saudi Arabia.

## Conclusions

In this study, we found that there was no relationship between delivery method and PPD. This study showed a higher prevalence of PPD in women with EMCS delivery. We also found that other factors such as chronic disease and education level may contribute to PPD. Accordingly, the Saudi private sector urgently needs to establish protocols and engage in public health promotion efforts focused on early detection and management in preventing such cases of PPD
